# Evaluation of the Quality of Otolaryngology Information on Persian Websites

**DOI:** 10.22038/ijorl.2019.31379.2031

**Published:** 2020-07

**Authors:** Ali Kouhi, Sasan Dabiri, Alireza Mohseni, Mohammadtaha Kouchakinezhad Eramsadati

**Affiliations:** 1 *Otolaryngology Research Center, Department of Otolaryngology, Head and Neck Surgery, Amir-Al’am Hospital, Tehran University of Medical Sciences, Tehran, Iran.*

**Keywords:** Google, Health, Internet, Quality

## Abstract

**Introduction:**

More and more patients are using the Internet to achieve information these days. Most patients (85%) use search engines to look for information about health. The quality of this information that patients encounter is highly different. This study aimed to assess the quality of information that an ear, nose, and throat patient would encounter when searching for information about their problem.

**Materials and Methods:**

The Persian keywords of most common otolaryngology problems were searched in Google. Moreover, the first 10 websites were selected by each search for the analysis using the DISCERN instrument. This instrument is made to evaluate the comprehensiveness and quality of health-related websites.

**Results:**

A total of 100 websites were evaluated in this study. However, 12 (12%) websites were excluded from further analysis due to copyright problems, advertisements, traditional treatments, and other reasons. The total DISCERN score for all 88 evaluated websites was obtained at 1.89 (SD=0.49). Moreover, the highest and lowest scores were 3.66 and 1.21, respectively. The search for “otitis treatment” had the highest results (mean DISCERN score=2.20, SD=0.38). The statistical analysis showed that the mean score for the Wikipedia.com Persian website was significantly higher, compared to the other Persian websites (P< 0.001).

**Conclusion:**

Persian websites have information with variable quality for the treatment of otolaryngology problems. Repeated websites, such as Wikipedia.com provided better information; however, the total quality of information was not satisfying.

## Introduction

The use of the Internet by patients to achieve information about health is growing these days. Before Internet development, access to this information was difficult for the public although books and medical journals were the only accessible resources (1,2). This information from the Internet increases the patients’ knowledge about their diseases and help them manage their disease better. In addition, this information encourages patients to participate actively in the treatment, do better in their medical decisions, and accept their physicians’ medical advice better (3).Nowadays, we have encountered information overloading problem due to high-speed information generation and distribution that has made it difficult for people to process the information (4). The majority of the patients (85%) look for health-related information and data by search engines with a wide range of quality (5). It is known that the quality of online information about health is a serious issue (6), and sometimes, the information found on the Internet may be harmful to patients’ health (7). Nowadays in our country, Iran, the number of people who use the Internet and Persian websites that provide health-related information has had a rapidly increasing growth. In 2011, the number of Internet users in Iran was 36,500,000 (out of a total population of about 75,000,000), which was more than 50% of all the people who use the Internet in the whole Middle East (8,9).Patients desire to look for online information in their language. In addition, online information has variable quality (10-14), and there is no supervision on Persian health-related websites which makes the quality evaluation of these websites necessary. Some previous studies have been performed to evaluate the quality of information about health on Persian websites in some fields (15-17); however, information quality has not been assessed in the field of otolaryngology in any previously conducted studies. Therefore, this study aimed to assess the online information quality which an otolaryngology patient would encounter while searching about their problem.

## Materials and Methods

This cross-sectional study was performed in 2016 on the Persian websites which provide health-related information, specifically for otolaryngology patients.

The websites were selected utilizing an Internet search engine, such as Google which is known as the largest and most popular Internet search engine. It is worth mentioning that more than two-thirds of Internet searches are performed using Google (18). In total, 10 common otolaryngology problems followed by the word "treatment" were typed in Google after translating into the Persian language (i.e., ear infection was translated into Persian language and entered into Google) (Table.1). Subsequently, the first 10 websites found by each search were selected for the analysis. However, the websites which were selling or advertising a product or providing only conventional medicine treatments did not undergo final analysis. After website selection, they were downloaded by the Scrap-book software for the Firefox Internet browser (available at https://addons.mozilla.org/en-US/firefox/addon/scrapbook/). Furthermore, the websites were downloaded at the same time and saved as offline files to prevent daily updates which disrupt the study procedure. The DISCERN was used to analyze each of the websites instruments (see http://www. discern.org. uk/discern-instrument.php). This instrument consists of 16 separate questions that are scaled from 1 which does not fulfill the question to 5 which completely fulfills the question. Information reliability is assessed in questions 1-8. Moreover, questions 9-15 assess particular features about the treatment options, and question 16 is a final quality scoring after considering the prior questions.

The data were analyzed in SPSS software (version 22.0) (IBM Corp., Armonk, NY, USA). The two-tailed student’s t-tests and one-way analysis of variance (ANOVA) were used to compare the DISCERN scores for the searched topics. A p-value less than 0.05 was considered statistically significant.

## Results

A total of 100 websites were found using Google search engine (10 websites for each common otolaryngology problem) (Table.1). However, 12 websites were excluded from the analysis with the DISCERN instrument since they had copied their content from other websites, or they were advertising a product or had conventional medicine content.

**Table 1:** Searched topics in Google search engine

**Searched Topics**	**Mean DISCERN Score**	**Standard Deviation**	**Number of excluded websites**
Ear infection	2.20	0.38	1
Sinusitis	2.09	0.55	1
Tonsillitis	1.75	0.45	2
Postnasal drip	1.94	0.73	1
Nasal congestion	1.77	0.43	1
Snoring	1.60	0.21	3
Hearing loss	1.66	0.20	1
Hoarseness	1.74	0.37	2
Sleep apnea	1.97	0.56	0
Ringing in ears	2.04	0.52	0

Websites, such as tebyan.ir, beytoote.com, and Wikipedia.com were found in different topic searches (Table 2). In total 60 different websites were found and evaluated in this study. These repeated websites made up 29 of 88 (32%) of the sites evaluated with DISCERN.

The mean DISCERN score was obtained at 1.89 (SD=0.49) for all the websites. In addition, the lowest and highest scores were estimated at 1.21 and 3.66, respectively.

**Table 2:** Repeated websites

**Website (frequency)**	**Mean DISCERN Score (SD)**
tebyan.ir (9)	1.96 (0.33)
beytote.com (5)	2.02 (0.47)
wikipedia.com (4)	2.75 (0.26)
hidoctor.ir (7)	1.74 (0.29)
vista.ir (4)	1.74 (0.29)

The ear infection and snoring treatments obtained the highest (2.20, SD=0.38) and lowest (1.60, SD=0.21) mean DISCERN scores, respectively (Table.1). According to the ANOVA test results, no significant difference was observed among the searched topics in terms of the DISCERN scores (P=0.172). The overall quality and completeness of information were far from satisfactory.

**Fig 1:**
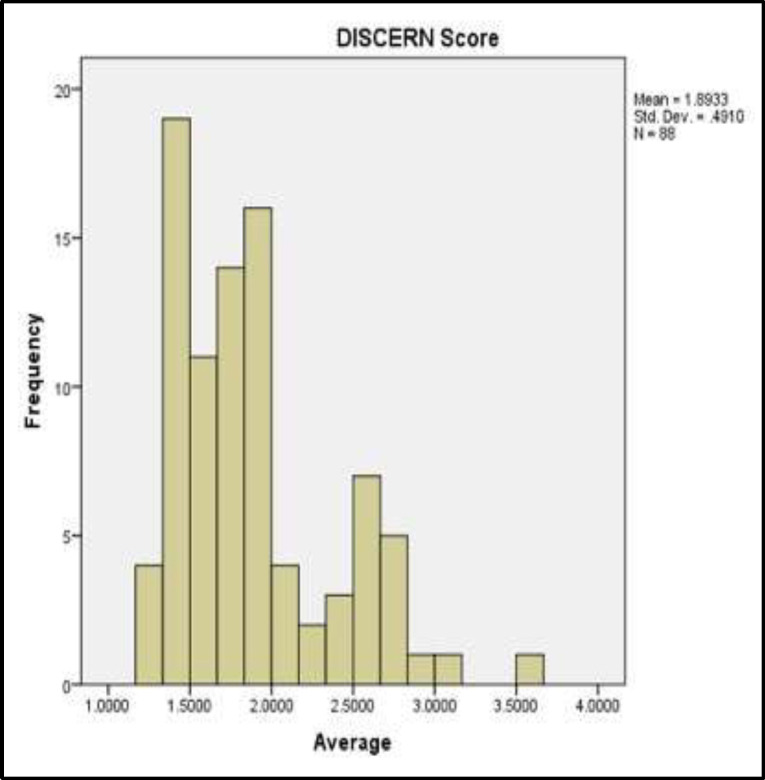
Histogram of the mean websites’ DISCERN score

 Out of 88 evaluated websites, 86 (97%) of them received scores less than 3, and 66 websites (75%) achieved scores less than 2 indicating the exceedingly poor quality of the information provided (Fig.1).

*Out of 88 evaluated websites, 86 (97%) of them received a score of less than 3. The majority of the websites achieved a score between 1 and 2 that indicates the exceedingly poor quality of the information provided.*

These sites missed elemental information about the source of data and did not bring up all treatment choices. No website received a score of 4 or higher that indicates a source of information with high-quality. Repeated websites (mean DISCERN score=2.01, SD=0.44) had higher scores, compared to the other websites; however, this difference was not statistically significant (P=0.495).

It was shown that the mean score achieved by the Wikipedia.com website was statistically higher, compared to other websites (P< 0.001). Furthermore, there was a statistically significant difference between the major websites (P=0.002).

The websites found for chronic diseases were compared with the ones for acute ones. In the same line, the results for more chronic disorders, such as tinnitus and sleep apnea were compared with the websites of more acute disorders, including ear infection. The lowest and highest mean DISCERN scores (SD) were 1.83 (0.48) and 2.02 (0.49), respectively. However, the difference was not statistically significant (P=0.098).

## Discussion

This study showed that the quality of Persian website information about the treatments of common otolaryngology problems is mostly dissatisfying. Moreover, the patients who look for online treatment information are exposed to a considerable amount of doubtful information. It was also revealed that repeated websites, such as Wikipedia.com had higher quality scores. These findings allow practitioners to monitor these repeated websites more precisely and direct patients to these websites for information about their condition.

Due to the increasing dependence of patients on information about health on the Internet, organizations, such as the Journal of the American Medical Association, has developed standards for assessing the quality of websites. These standards are useful; however, it has been shown that websites meeting these standards can still be full of inaccurate information (7). This adds to the complexity and confusion while evaluating websites for their information.

One of the main problems in analyzing the quality of online information is that its accuracy is not warranted. The DISCERN instrument gives us the means to assess the quality of treatment options; nonetheless, the evaluation of the information accuracy is missed, which is the drawback of this tool since it needs high quality and highly accurate information. As warranting accuracy is difficult, practitioners should inform patients of this fact that although some websites are better than others, they may still have the wrong information. This seems more important when other studies declare that most of the patients (nearly two-thirds) do not share the health information they find online with their physician (19,20). Patients who search the Internet to achieve information about their disease are rapidly growing in the number. Due to the variations in quality and accuracy of the websites that give health-related information, physicians should help the patients in their searches and guide them in their decisions. Accordingly, physicians must find websites that are accurate and valuable in their related fields.

Although this study paved the way for the evaluation of the quality of otolaryngology information on Persian websites, it suffers from some limitations. First, it was aimed to imitate the method that an otolaryngology patient may try to find information about the common problems. The selected words were simple and ordinary Persian language keywords for common otolaryngology conditions that were thought to be the most probable terms that most patients would use. It is obvious that there are several ways to explain these keywords, and each of them may have little different outcomes. 

Moreover, the Google search engine was used in this study since there are reports that it is used by the majority of people (18). However, other search engines would produce different results. Google uses search algorithms that are based on the search location. Therefore, the search outcomes may have variations in various districts of the country. Regardless of the probable bias and error, it is believed that the repeated websites would be included in the searches of a great majority of patients, and therefore, the conclusion of this study that these websites give the patients more trustworthy information would be true.

## Conclusion 

This study showed that the quality of online health-related information for the treatment of otolaryngology problems is below ideal. The repeated websites, such as Persian Wikipedia had a statistically higher quality, compared to other websites; however, the total quality of information was not acceptable. Further studies are needed to focus on the effects of the searches on patients’ health and medical condition.
